# Human Parvovirus 4 Viremia in Young Children, Ghana

**DOI:** 10.3201/eid1810.111836

**Published:** 2012-10

**Authors:** Jürgen May, Jan Felix Drexler, Ulrike Reber, Nimarko Sarpong, Ohene Adjei, Marcus Panning, Christian Drosten, Anna Maria Eis-Hübinger

**Affiliations:** Bernhard Nocht Institute for Tropical Medicine, Hamburg, Germany (J. May);; University of Bonn Medical Centre, Bonn, Germany (J.F. Drexler, U. Reber, C. Drosten, A.M. Eis-Hübinger);; Kwame Nkrumah University of Science and Technology, Kumasi, Ghana (N. Sarpong);; Kumasi Centre for Collaborative Research in Tropical Medicine Kumasi (O. Adjei);; and Freiburg University Medical Center, Freiburg, Germany (M. Panning)

**Keywords:** human parvovirus 4, PARV4, viruses, human partetravirus, genotype 3, children, blood, plasma, whole blood, viremia, Africa

**To the Editor:** Establishment of viremia is a characteristic feature of infection with human parvovirus 4 (PARV4). In northern Europe, PARV4 (human partetravirus) is primarily transmitted by blood-borne routes (*1*,*2*). In other areas (southern Europe, western Africa, South Africa, Asia) infection seems to be more widespread, suggesting alternative modes of virus acquisition (*3*–*6*).

We reported PARV4 genotype 3 viremia in young children with unknown parenteral blood exposure from the rural Ashanti region of Ghana (*7*). In that study, 2 (2.1%) of 94 children (median age 14.9 months) and 22 (11.9%) of 185 children (median age 24.0 months) were virus positive (ages of the 2 virus-positive children from the younger cohort 14.9 and 15.6 months). Because the number of infants was small in that study, we extended our investigations on PARV4 viremia to an additional cohort of 15-month-old children from the same study group.

Plasma samples from 361 randomly selected children (191 girls) were tested. Specimens were collected during January–December 2004 during a trial of intermittent preventive malaria treatment in the rural Afigya Sekyere District, Ashanti Region, Ghana (*7*). Plasma samples were analyzed because of limited availability of whole blood samples. Median age of children was 14.9 months (range 13.8–17.5 months, interquartile range 14.5–15.2 months).

Nucleic acid was prepared from 200-μL plasma samples by using the NucliSENS EasyMAG system (bioMérieux, Nürtingen, Germany). All samples were analyzed by using 2 real-time PCRs and primers described elsewhere (*7*,*8*). The limit of detection was ≈200 plasmid copies/mL. Strict precautions were applied during plasma handling and amplification to avoid false-positive results.

PARV4 genotype 3 DNA was detected in plasma of 32 (8.9%) of 361 children. Viral load ranged from ≈200 copies/mL to 3.0 × 10^4^ copies/mL (Figure). Median viral load was 453 copies/mL. Nucleotide sequencing of screening PCR amplicons and additional genomic regions amplified from 6 plasma samples identified the viruses as PARV4 genotype 3 (GenBank accession nos. JN183933–JN183938). There was no association between history of fever, anemia, or erythema in children with or without PARV4 viremia (p>0.05, by χ^2^ test).

**Figure:**
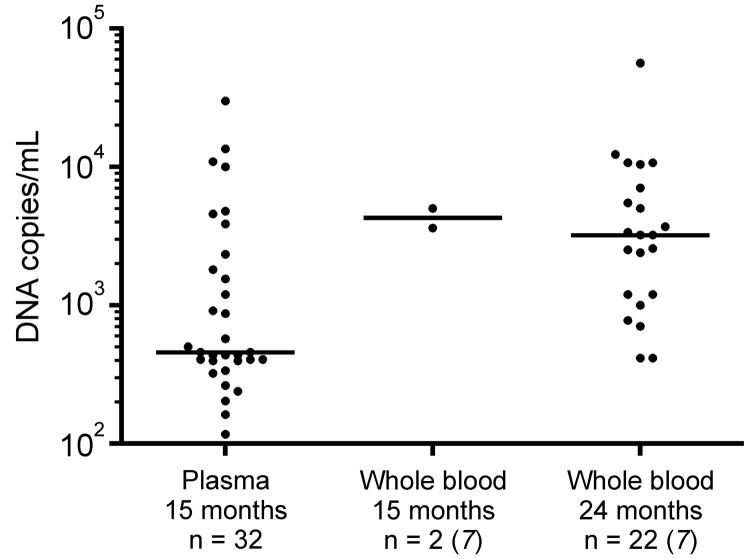
Parvovirus 4 DNA loads in virus-positive plasma specimens from children compared with those in whole blood samples previously tested (*7*), Ghana. Virus concentrations are given on a log scale on the y-axis. Each dot represents 1 specimen. Horizontal lines represent median values for each sample group. Children whose plasma was tested had a median age of 15 months, and children whose whole blood was tested had a median age of either 15 or 24 months. Viral load data (i. e., median viral load and range) for the 2 groups of whole blood samples have been reported (*7*) and were included for comparison with plasma data from this study.

PARV4 viremia status was already known for 78 children 24 months of age (*7*). These data enabled comparison of viremia at 2 time points (24 months and 15 months of age). Of these 78 children, 10 had viremia (viral load range 4.0 × 10^2^–1.4 × 10^4^ copies/mL) and 3 (3.8%) showed viremia at both time points and identical viral nucleotide sequences (time between bleedings 8.7 months for 2 children and 9.0 months for 1 child). However, only short genomic regions (780 nt for 1 child, 599 nt for a second child, and 95 nt for a third child) could be amplified and sequenced because of low viral loads. Four children had positive results in the first sample, and 3 had positive results in the second sample.

Because comparison of large and contiguous parts of the viral genomes within each sample pair was not possible and serologic data were lacking, PARV4 positivity over a 9-month period can be interpreted by 3 hypotheses. First, detection of PARV4 DNA over time might represent long-term viremia after infection, similar to observations in human parvovirus B19 infection. Second, demonstration of PARV4 during widely spaced intervals might indicate endogenous reactivation of viremia. Third, exogenous reinfection might have occurred.

PARV4 viremia was detected in a study in the United Kingdom among 110 PARV4-negative persons with hemophilia screened over 5 years for PARV4 viremia and seroconversion (IgG and IgM) (*9*). Nine patients who seroconverted were identified, and 1 had PARV4 viremia (genotype 1) over an 8-month period. Viral loads for this patient were low (<10^3^ copies/mL), a finding similar to ours for the 3 children. However, negative IgM results in the person with hemophilia suggest that the sampling window might have missed the acute infection.

Comparison of results of our study with those of our previous study (*7*) showed 2 differences. First, frequency of viremia in children tested previously at 15 months of age was lower (2.1%, 2/94) than in the children in this study (8.9%). Second, median viral loads differed by nearly 1 log_10_, with the higher concentrations in the previous study analyzing EDTA whole blood. Whether these differences were caused by the relatively small number of children included or by the fact that whole-blood samples were compared with plasma samples remains to be clarified. However, our previous hypothesis that prenatal or perinatal transient infection was an unlikely mode of virus acquisition needs to be modified because PARV4 infection in newborns has recently been demonstrated (*10*). 

Although we lacked IgM and IgG serologic data to interpret our findings, our study suggests that PARV4 genotype 3 infection might be characterized by viral persistence, reactivation, or reinfection. Additional longitudinal studies, including serologic testing for short intervals, are needed to determine the pathogenesis and potential public health role of PARV4 infection.
